# Defect Characteristics and Online Detection Techniques During Manufacturing of FRPs Using Automated Fiber Placement: A Review

**DOI:** 10.3390/polym12061337

**Published:** 2020-06-12

**Authors:** Shouzheng Sun, Zhenyu Han, Hongya Fu, Hongyu Jin, Jaspreet Singh Dhupia, Yang Wang

**Affiliations:** 1 School of Mechatronics Engineering, Harbin Institute of Technology, No. 92, Xidazhi Street, Harbin 150001, China; szsun@hit.edu.cn (S.S.); hanzy@hit.edu.cn (Z.H.); hongyafu@hit.edu.cn (H.F.); wyyh@hit.edu.cn (Y.W.); 2 Department of Mechanical Engineering, University of Auckland, 20 Symonds Street, Auckland 1010, New Zealand

**Keywords:** automated fiber placement, manufacturing defects, mechanical properties, control strategy, online detection techniques

## Abstract

Automated fiber placement (AFP) is an advanced manufacturing method for composites, which is especially suitable for large-scale composite components. However, some manufacturing defects inevitably appear in the AFP process, which can affect the mechanical properties of composites. This work aims to investigate the recent works on manufacturing defects and their online detection techniques during the AFP process. The main content focuses on the position defect in conventional and variable stiffness laminates, the relationship between the defects and the mechanical properties, defect control methods, the modeling method for a void defect, and online detection techniques. Following that, the contributions and limitations of the current studies are discussed. Finally, the prospects of future research concerning theoretical and practical engineering applications are pointed out.

## 1. Introduction

**Figure 1:**
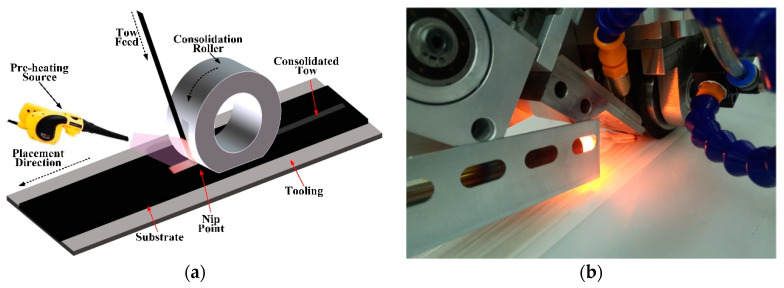
The automated fiber placement (AFP) working principle and AFP machine produced by Coriolis: (**a**) AFP working principle [22], (**b**) AFP machine produced by HIT.

Fiber reinforced plastics/composites (FRPs) not only have many advantages, including a light weight, high strength, and high temperature resistance, but can also be used to integrate and manufacture large-scale integral components. They are superior to traditional metal materials (e.g., steel and non-ferrous metals) in terms of weight reduction, fatigue resistance, corrosion resistance, reliability, and maintainability, and are becoming more widely used in aerospace, transportation, energy, and defense, etc. [1,2,3,4] The manufacturing method of FRPs includes hand layup, molding, pultrusion, filament winding, automated tape laying, and automated fiber placement. Among them, automated fiber placement (AFP) appeared in the 1970s in the aerospace industry. It combines the advantages of filament winding and automated tape laying to overcome their limitations and exploit their benefits, and is one of the fastest-growing and most effective fully automated manufacturing technologies for composite materials in recent years [5,6,7,8,9,10,11,12]. AFP technology can be used not only for producing thermosetting or thermoplastic composites but also for dry fiber placement [13,14,15]. An AFP machine usually consists of a placement head and functional mechanical structure (a robotic arm or gantry structure). The placement head lays prepreg tows onto a mold to construct the layup. Depending on the shape of the mold surface, the AFP process can use multiple prepreg tows (8~32 tows) to realize continuous variable angle placement. Additionally, it can cut/refeed the tow to adapt to the change of the manufacturing boundary, which can guarantee the processing requirements of complex structures [16,17,18,19,20,21,22,23]. The AFP working principle [22] and AFP machine produced by Harbin Institute of Technology (HIT) are shown in Figure 1.

Due to the complexity of the AFP manufacturing process, especially multiple process parameters, prepreg defects, and manufacturing errors, the laminates are not exempt from imperfections, such as gaps and/or overlaps, twisted tows, fiber waviness, and air pockets, which often appear in the final component, thereby affecting the mechanical performance [24,25,26,27,28,29,30,31]. The contribution of literature [32] has been to classify the defects during the AFP process, including the following four categories:

- Positioning defects (gaps, overlaps, missing tows, twisted tows, etc.);
- Bonding defects (bridging, air pockets, etc.);
- Foreign bodies;
- Tow defects.

**Figure 2:**
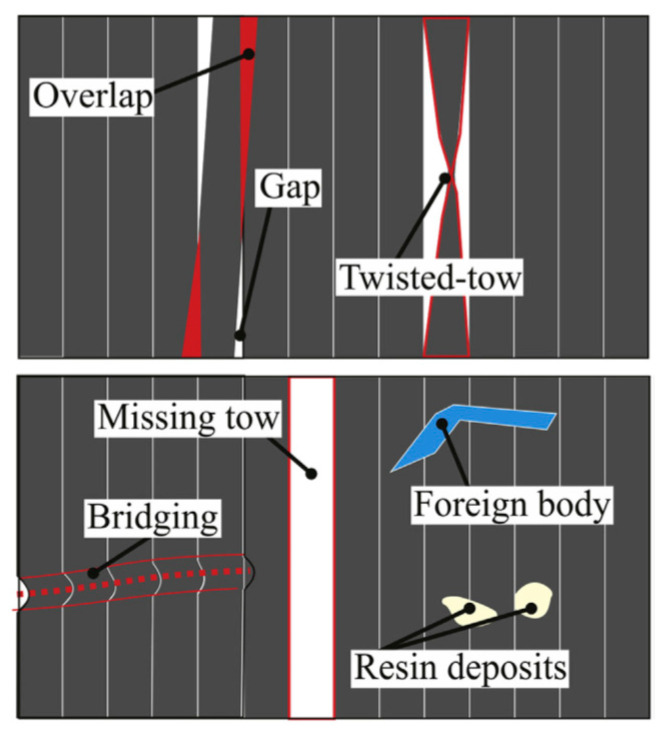
Defects occurring during the automated fiber placement process [32].

The different types of defects are shown in Figure 2.

For the AFP process, only the first two types of defects are controllable by AFP process optimization. Thus, this paper focuses on the positioning defects and bonding defects to discuss the current research progress and existing problems. The rest of this paper is organized as follows. In Section 2, the positioning defects during the AFP process, mainly gaps and overlaps, are introduced regarding conventional laminates and variable-stiffness laminates, respectively, wherein the mechanical properties of the laminates under different defect levels and defect-controlling process techniques are discussed. Section 3 focuses on the modeling method of void defects for the thermoplastic composites made by the AFP process. In Section 4, the existing online detection techniques for the defects are enumerated. Some novel detection techniques are investigated, including an optical fiber Bragg grating method, a thermal image processing method, and a stress wave method. Also, some advantages and disadvantages of the detection methods are further discussed. Finally, the research prospect of the defects during the AFP process is pointed out.

## 2. Positioning Defects

### 2.1. Conventional Laminates

**Figure 3:**
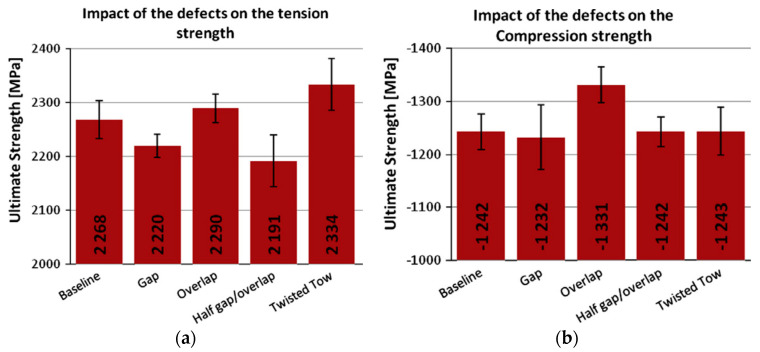
The effect of the defects on some strengths: (**a**) defects and tension strength, (**b**) defects and compression strength [35].

In this paper, conventional laminates refer to directional and unidirectional laminates. In manufacturing complex shapes or surface parts, misalignments can appear on the band edges or the adjacent tows inevitably, such as gaps, overlaps, fiber waviness, twisted tows, and bridging, etc. For intraply gaps and overlaps, Sawicki et al. [33,34] pointed out that the fiber placement is easier to exhibit in in-plane waviness than tape laying. Due to the width variance of tows caused by in-plane waviness this can form gaps and/or overlaps around in-plane waviness, which can cause out-of-plane waviness in adjacent plies. The relationship between the defects and compression strength was also investigated. The results show that compression strength is reduced 15−20% when overlaps and gaps from 0.03” to 0.12” wide are laid. The rate of decrease in compressive strength is significant for gap sizes smaller than 0.03”, but the strength reduction is relatively constant for larger gap sizes. Also, they found that the decrease in the compressive strength of the unnotched and notched laminates was the same. Croft et al. [35] performed some experimental works to investigate the effect of defects on ultimate strengths, including gaps, overlaps, half gap/overlap, and twisted tows. The strengths were tested at the lamina level (fiber tension, fiber compression, and in-plane shear), as well as at the laminate level (open hole tension (OHT) and open hole compression (OHC)). Each test was compared with a baseline configuration without defects, which is shown in Figure 3 and Table 1.

The results showed that the defects did not always lead to a reduction in mechanical performance. Generally, a defect that gives an improvement in one mechanical performance also penalizes another performance. Therefore, some defects could be selected to be avoided or controlled based on the special mechanical requirements of some parts.

Marrouze et al. [36] proposed an advanced computational multiscale and multiphysics damage tolerance approach in order to evaluate the effects of defects, which combined micromechanics with the finite element method, damage tracking, fracture, and property prediction with and without defects. The approach was verified by testing experiments. According to the simulations, this paper obtained the following important conclusions. The initial gaps reduce the strength of notched laminates, but the effect diminishes as the gap size grows. This conclusion is similar to the compressive strength results in [34]. It is found that gaps in stiffened panels have minimal effect on stability and strength. Also, their reasons for the formation of fiber waviness were further discussed. When another ply is laid on the laid ply where the gaps exist fiber waviness is introduced into the gaps. The degree of the waviness is driven by the height of the gap that depends on the tow thickness but not the gap length.

Lan et al. [37] investigated the effect of embedded defects on the mechanical properties of composites made by the AFP process. Two stacking sequences, [(−45°/+45°)_3_/−45°] and [90°_4_/0°_3_/90°_4_], have been examined, either with or without a caul plate, in which gaps and overlaps were introduced. According to the ultrasonic C-scan, in-plane shear tests, and compression tests, the results of ±45° laminates indicated that the presence of defects in two adjacent plies could limit the healing resulting in resin-rich areas for gaps and fiber-rich areas for overlaps. But these effects can be reduced by curing with a caul plate. The effect of gaps on in-plane shear properties is more significant than that of overlaps. For 90°/0° laminates, the curing process plate allows partial healing of defects without a caul while complete healing with a caul plate. Further, no caul plate can result in the strong effect of defects on compression properties. In this way, the use of a caul plate limits this failure mechanism.

Guin et al. [38] examined the effect of gaps by experimental tests. Although similar to the research in the literature [35], this paper pays more attention to the application in the aerospace industry. The laminates with the stacking sequence [+45/−45/90/0]_2s_ using IM7/8552-1 prepreg were made by the AFP process. Then, the laminates were tested in unnotched tension, unnotched compression, and open hole compression. The results show that the primary effect of gaps is fiber waviness. The combination of tensile loading and significant fiber waviness has a significant effect on strength but no effect on modulus. Compression loading serves to exacerbate the degree of fiber waviness, while tension loading serves to alleviate it. So the axial stiffness in compression is adversely affected in the gaps. Interestingly, the relative reduction in notched compression strength is less significant than that in unnotched compression.

Woigk et al. [39] investigated the effect of gaps and overlaps on tensile and compression properties experimentally. Specimens were made by the AFP with a quasi-isotropic, symmetric layup into which artificial defects can be induced in various defined formations. Four defect configurations were developed, including “Gaps”, “Overlaps”, “Staggered Gaps”, and “Gaps and Overlaps”. It can be concluded that “Gaps and Overlaps” specimens exhibit strength reductions in tension and compression of 7.4% and 14.7%, respectively.

The fatigue of the laminates is also an important mechanical performance [40,41,42,43]. Elsherbini et al. [44] investigated the effect of gaps on the fatigue behavior of unidirectional carbon/epoxy laminates. Tension-fatigue tests were performed with defected samples and then compared to defect-free samples. The infrared thermography technique was used to monitor the propagation of damage during fatigue loading. Furthermore, a fatigue progressive damage model (FPDM) was developed to predict fatigue damage progression and the failure of laminates, which has a good agreement with experiments. The experiment also showed that the effect of gaps can depend on the maximum applied stress during fatigue.

The works on conventional laminates in this review are summarized in Table 2.

### 2.2. Variable-Stiffness Laminates

#### 2.2.1. Defect Control Method

**Figure 4:**
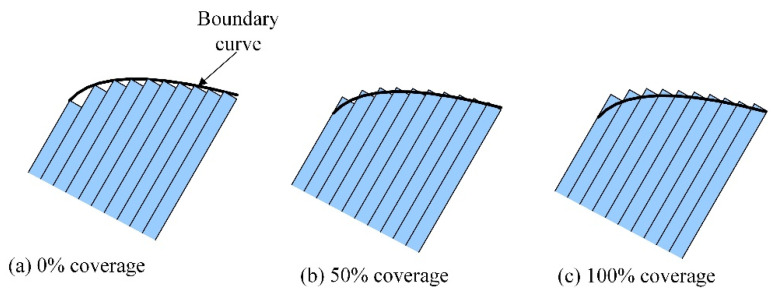
Tow dropping with different coverage parameters adapted from the reference [52].

An important advantage of the AFP process is the possibility of making variable-stiffness laminates with curvilinear fiber paths to strengthen the structural buckling performance of composites [45,46,47,48,49,50,51]. However, to manufacture variable-stiffness components, since the tow has a certain width while translation distance remains constant, some imperfections are easier to form between the adjacent tows, mainly gaps and/or overlaps. For solving the problem above, some methods have been developed. Among them, the tow-drop method is an effective method to reduce defects. This technique uses the cutting/refeeding function of an AFP machine to reduce or even eliminate overlapping areas by controlling the number and length of tows. In reality, however, if a constant thickness is desired, tows will be cut perpendicularly to the fiber direction, resulting in small triangular resin-rich areas without fibers, as seen in Figure 4a. Thus, different coverage parameters can be used, as in Figure 4b,c. A 0% coverage exhibits a small triangular resin-rich area. Similarly, if the full coverage method (100% coverage) is used, the overlaps can be induced on the surface of the laminate, resulting in a rough surface. Therefore, Blom et al. [52] pointed out that the course boundaries were assumed to be smooth, which is not accurate regarding the references [47,48,49,50,51,52,53,54,55,56,57,58].

Furthermore, they used the finite element method to study the effect of tow-drop areas on the strength and stiffness of a variable-stiffness laminate, in which some factors were considered, including tow width, laminate thickness, and staggering. It can be concluded that damage is initiated at tow-drop areas, especially in regions with high fiber orientation angles. Based on numerical studies, staggering improves the strength of a laminate, but there is no apparent relationship between the strength of tow-drop laminates and the laminate thickness. As mentioned above, staggering [59] is also a better method to reduce the effect of defects on the mechanical properties of laminates to some extent. Staggering is the shifting of plies with the same orientation concerning each other. Thus, course boundaries, overlaps, and gaps of repeated plies do not occur at the same planar location. The basic principle is that if two repeated plies are present in the laminate, they will be shifted by the half distance between two-course centerlines. This way, repeating four times means that one quarter of the course shift is needed [54,58].

**Figure 5:**
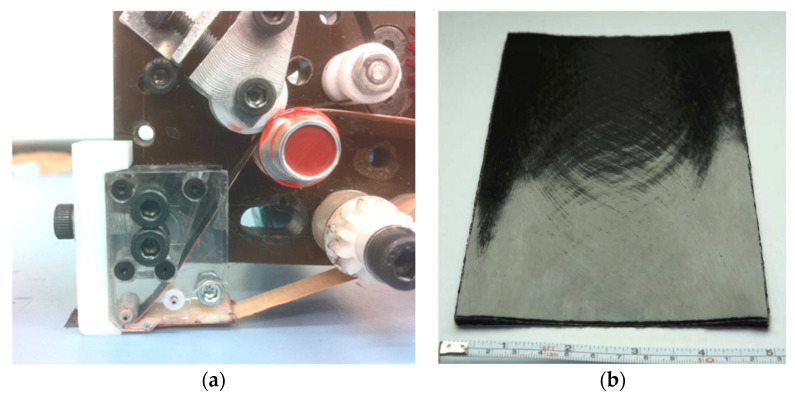
The Continuous Tow Shearing (CTS) technique: (**a**) laying head [60], (**b**) specimen made by CTS [61].

Given the shortcomings of traditional methods for limiting defects, Kim et al. [60,61,62,63] developed a novel Continuous Tow Shearing (CTS) technique. In the conventional AFP, defects are mainly caused by the in-plane bending deformation of the tow. The AFP head follows the curved tow path, rotating its vertical axis so the fibers inside and outside the reference tow path inevitably buckle and stretch. Thus, a special CTS laying head was designed to utilize the in-plane shear deformation of the tow inherently, as shown in Figure 5a. This technique significantly reduces the design limitations of the fiber radius of curvature and there are no gaps or overlaps between the tows. The quality of laminates produced by CTS is significantly higher than with traditional techniques. But the thickness of the laminate changes with the variation of the fiber angle due to changes in tow width (see Figure 5b).

#### 2.2.2. Defect and Mechanical Properties

To obtain the relationship between the defects and the mechanical properties in variable-stiffness laminates, Fayazbakhsh et al. [64] introduced a defect layer method which can be a regular composite with embedded defects. The method uses a finite element model to capture the effect of gaps and overlaps with a lower number of elements compared to the approach in [52], which enables us to calculate gaps and overlaps precisely. The results showed that gaps can deteriorate in-plane stiffness and buckling load, but overlaps can improve structural performance. For the effect of gaps with the laminate configuration of [±(43, 0.48)/±(44, −1.57)/±(35, −1.57)/±(38, −1.57)]_s_, the improvement of the bulking load resulting from fiber steering decreases to 20%. However, overlaps increase the improvement of the buckling load to 78%. Even in the laminate configuration of [±(43, 0.48)/± (48, −1.57)/±(30, −1.57)/±(26, −1.57)]_s_ overlaps could improve the in-plane stiffness and buckling load to 11% and 71%, respectively. In some laminate configurations, the overlap can compensate for the effects of the gap. Similarly, Wu et al. [65] conducted experiments and showed that the buckling stiffness of a variable stiffness laminate with overlaps is 27% higher than a cross-ply laminate with [±45]_5S_, but it is only 4% higher for the variable stiffness laminate with gaps.

**Figure 6:**
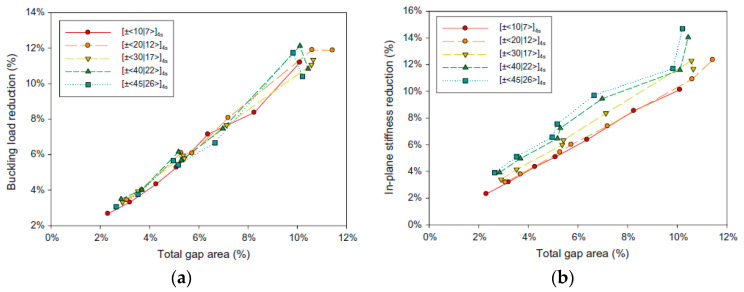
The effect of gap area percentage on the structural performance of variable stiffness laminates: (**a**) the buckling load, and (**b**) in-plane stiffness [66].

Based on the previous work of Fayazbakhsh [64], Nik et al. [66] investigated the effect of design parameters (curvilinear fiber path) and manufacturing parameters (tow width, the number of tows in a course, and tow strategy) on the gap and overlap area percentages within variable stiffness. The buckling load and in-plane stiffness in defected laminates were then calculated using Pareto solutions of variable stiffness. From this paper, it is seen that the largest number of tows with the smallest width can lead to the minimum defect area percentages. For instance, a course with 32 tows, each 3.175 mm wide can reduce the number of defects by two-thirds compared to a course with 8 tows, each 12.7 mm wide. Some results are shown in Figure 6.

Following the previous work of Blom [52], Falco et al. [67] conducted some experiments to investigate the effect of the fiber angle discontinuities between different courses in a ply on the unnotched and open hole tensile strength of the laminate. Different manufacturing strategies were studied in detail, including tow-dropping with 0% gap coverage, tow-dropping with 100% gap coverage, and tow-dropping with 0% gap coverage and ply staggering. In comparison with the baseline specimens (a straight fiber panel without defects), the result showed that 0% gap coverage and ply staggering could be expected to be an effective combination to reduce the effect of defects in variable stiffness laminates. The testing experiments also showed that large delaminations could usually initiate around the tow-drop defect area, leading to matrix cracking and finally fiber failure, which was probably caused by the amplification of the interlaminar stresses around the defects. The remote failure stress for un-notched tensile (UNT) and open-hole tensile (OHT) specimens are shown in Table 3.

**Figure 7:**
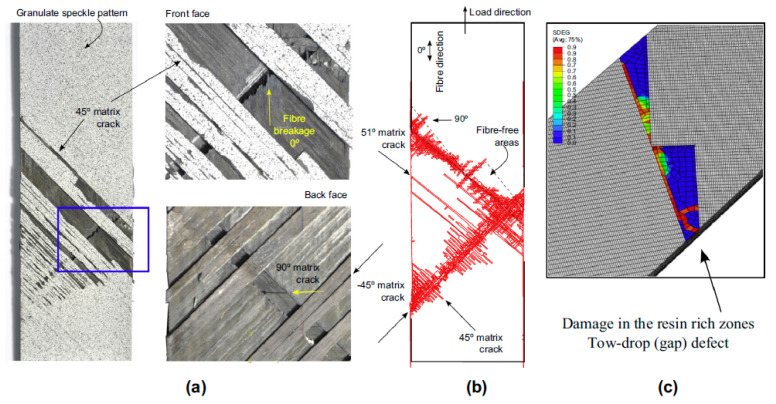
A comparison of simulation and experimental results: (**a**) progressive intralaminar damage for a real “UNT-gap” coupon after the experimental test [67]; (**b**) superposition of the intralaminar failure mechanisms predicted through the virtual test; (**c**) undeformed damage prediction in the resin zone [68].

Furthermore, Falco et al. [68] developed a reliable mesoscale virtual testing approach to investigate the effect of tow-gap effects on the mechanical performance of notched and un-notched specimens under in-plane tensile loads. X-ray computed tomography (XCT) was used to observe the fiber angle discontinuities between different tow-courses in a ply and the tow-gap distributions. Then, finite element analysis, coupled with different constitutive models for the fiber-reinforced material, resin-rich areas, and ply interfaces was performed and applied to a three-dimensional domain. Some results, such as the tow-drop effects on plain and notched laminates, were validated in previous work [67]. Thus, the prediction results using the mesoscale virtual testing approach can be seen as accurate, as shown in Figure 7. However, tow overlaps were not considered in this paper.

Li et al. [69] pointed out that out-of-plane waviness by various combinations of gaps and overlaps were less studied [52,55,56,64,66,70]. Thus, they developed 3D meshing tools to automatically generate ply-by-ply models as well as out-of-plane waviness and ply thickness variations caused by gaps and overlaps, in which it is easy to create different defect combinations to investigate the effect of defect size and distribution on the strength knockdown. The models also can predict the reduction of strength as a function of the magnitude and type of defects. However, for more complex models, further experimental validation is required.

**Figure 8:**
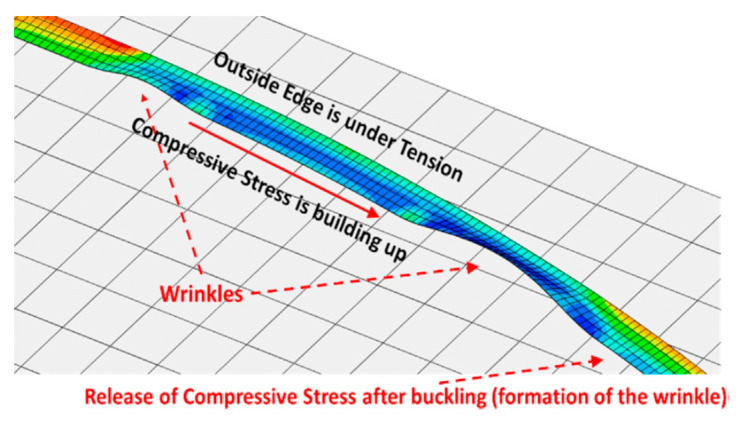
Stress distribution in a common place result in the simulations of defective layups [83].

Some studies [71,72,73,74,75] indicated that prepreg tack resists the formation of most layup defects, which serve as a parameter to evaluate a strong intimate contact, stress-relaxation effects in the prepreg resin, and the resin’s cohesive strength. Further, tack is controllable by process parameters, especially pre-heating temperature. The works of [74,76] investigated the tack, peel resistance, and bonding issues during the AFP process. The effect of compaction force, laying speed, and temperature on the adhesive properties of tow prepreg was studied on the ply-tool interface. The results showed a strong temperature effect, with the prepregs requiring a higher layup temperature to accommodate higher layup speeds. The predicted peeling forces were in agreement with the experiments. However, the effects of other parameters such as the tool’s surface roughness, resin, and the tool surface energies on prepreg tack are not very well understood. Additionally, researchers [77,78,79,80,81,82] have used simulations and many experiments to tackle the problem of wrinkle formation during tow steering. However, these methods are expensive and time-consuming. Thus, Bakhshi et al. [83] proposed a novel modeling strategy considering the prepreg laying process and the prepreg tack. Firstly, AFP experiments were performed using different process parameters identified as the five major types of defects, including in-plane fiber waviness, sheared fibers, tow pull-ups, blisters, and out-of-plane wrinkles. According to some patterns and conditions of defect formation, a new global defect modeling method to model the AFP process was presented, as shown in Figure 8. Then, the surface-based cohesive zone modeling technique using a bilinear traction-separation law was used to model the prepreg tack. A comparison of the simulation results with the experiments showed an excellent agreement in the patterns and frequencies of defects.

Some researchers have studied the vibration of the laminate [84,85,86,87,88]. For the relationship between the vibration and defects, Akbarzadeh [89] et al. examined the vibration of fiber-steered plates with a sandwich structure made by the AFP. Third-order shear deformation theory, the hybrid Fourier–Galerkin method, and the numerical integration technique were used to predict the vibration responses under manufacturing defects, particularly gaps and overlaps. Furthermore, they used the magnetostrictive layers to suppress the structural vibration of the laminates. The results of the vibration analysis showed that the dynamic out-of-plane deflection in the plates with gaps had a higher amplitude and a lower frequency than that of a defect-free plate. Additionally, the magnetostrictive layers can lead to a lower vibration frequency, and better attenuate the vibration response of the plate.

The works on variable-stiffness laminates in this review are summarized in Table 4.

## 3. Void Defects

Voids are also common defects in laminates which can significantly affect the mechanical properties of composites [90,91,92]. The small bubbles in the prepreg itself, the volatilization of the resin in the pre-heating process, low laying pressure, and small air pockets in the laying process can result in the initial bubbles. For thermoplastics, the voids generated during the laying process are the final defects due to the in-situ curing technique. For thermosetting plastics, the defects before curing can significantly affect the formation of voids after curing [93,94,95,96,97]. Their formation mechanisms are entirely different from the positional defects. Voids and their distribution have an adverse effect on the interlaminar stress and the mechanical properties of the fiber/matrix interface, especially the interlaminar shear, compression, and bending properties [98,99,100,101,102]. There have been a great number of studies focusing on the relationship between the voids and the properties of composites. For instance, Judd et al. [103] obtained experimental results which showed that the interlaminar shear strength decreases by 7% for every 1% increase in void content when the void content is less than 4%. Hagstrand et al. [104] experimentally found that voids have an adverse influence on flexural modulus and strength. Each 1% increase in void content results in a 1.5% reduction of the properties before the void content reaches 14%.

For the characteristics of voids during the AFP process, Ranganathan et al. [105,106] proposed a novel approach to model the in-situ tow placement process of thermoplastic composites using a Newtonian fluid in a two-dimensional geometry, which is capable of predicting the final void content and the thickness of a composite part as a function of the laying speed and compaction pressure under non-isothermal conditions. Similarly, Tierney et al. [107] developed series of integrated sub-models, including a heat transfer model, void dynamics model, and multi-scale void transport model, to predict the heat transfer and void dynamics within the laminate. Furthermore, experiments were conducted, and the results showed that significant gradients in the final void content exist through the thickness and are directly related to the processing conditions of the heating temperature, laying speed, and torch height. Simacek et al. [108] established a dynamic change model of the voids of thermoplastic composites made by the automated placement technique. The model combines the internal pressure of bubble, the pressure of compaction roller, the response of fiber matrix and the pressure of resin to study the process of the resin diffusing from the resin-rich region to fill the bubble. Khan et al. [109] developed a simulation tool from the existing available model in the literature [105,106,107]. The effects of consolidating the force, laying speed, and hot gas flow in the heating region on the void development were then investigated through simulation. Furthermore, experiments were carried out to manufacture some AS4/PEEK (a grade of thermoplastic composites) laminated plates. The void distributions’ through-thickness and density were compared with the experimental values. The results showed that the simulation method is effective. However, there are not many studies on voids in the automated manufacturing process.

**Figure 9:**
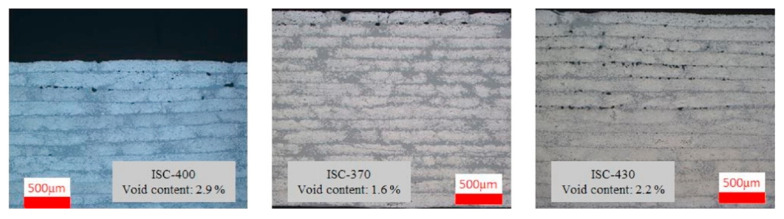
Cross-section optical micrographs of samples manufactured in AFP with in-situ consolidation (ISC) [110].

Recently, Seanz-Castillo et al. [110] studied the effect of process parameters on voids and the mechanical performance of CF/PEEK composites. They used three different out-of-autoclave technologies, including vacuum bags, hot-press, and thermoplastic automated fiber placement (TP-AFP) with in-situ consolidation (ISC). The void characterizations were performed using the density method, matrix acid digestion, 2D microscopy, and C-scan, thus summarizing the benefits and scope of various methods. The results show that ISC voids focus mainly in the upper laminates (see Figure 9). The reason for this is that the bottom layers can suffer more rolling times from the compaction roller. This conclusion can inspire us to press a few more times by roller onto the surface of the laminates after the TP-AFP process. The disadvantage of this paper is that only the effect of temperature is considered during the TP-AFP process, but other process parameters are not considered.

Additionally, few researchers have examined the voids of thermoset materials during the AFP process. Although the bubbles in the laminates during the AFP process are not final defects, the work of [111] indicates that there is a specific relationship between the bubbles in the laminate before curing and the voids after curing. Therefore, the bubbles in the thermoset laminates during the AFP process should also be studied.

## 4. Online Defect Detection Techniques

**Figure 10:**
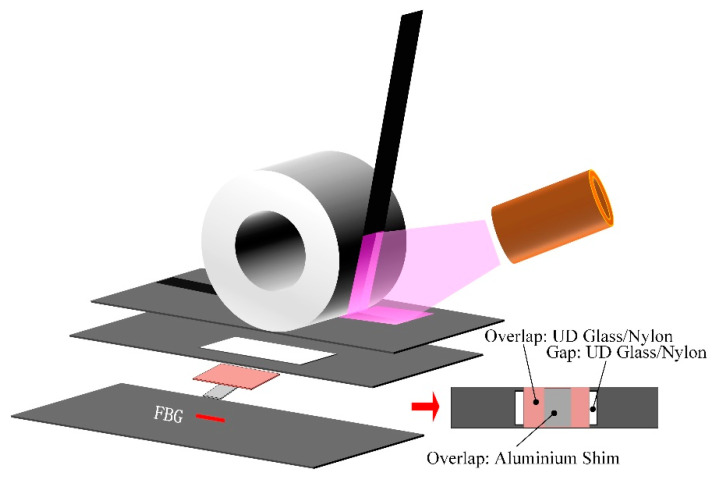
The configuration of defect-induced laminates manufactured using AFP adapted from the reference [122].

To detect the defects during the AFP process, some online defect detection techniques have been developed [112,113,114,115,116,117,118,119,120,121], such as machine vision, digital image processing, thermographic monitoring, and optical sensors. Among them, some novel detection methods are introduced here. Oromiehie [122] et al. used optical fiber Bragg grating (FBG) sensors to identify the misalignment defects during the AFP process. Four specimens were made by AFP, including defect-free artificial gaps and overlaps, and overlaps induced by aluminum shim, as shown in Figure 10. Through reflected wavelength changes, the type of defects in terms of size and materials can be identified. The results showed that the FBG sensors could be reliably implemented for online defect monitoring during the AFP process. However, for small actual defects under the micro scale, such as air pockets, the applicability of the method needs to be further confirmed. Additionally, another problem is that it is inconvenient to take the sensor out from the preform before the curing process, so this method has not yet been industrially applied.

**Figure 11:**
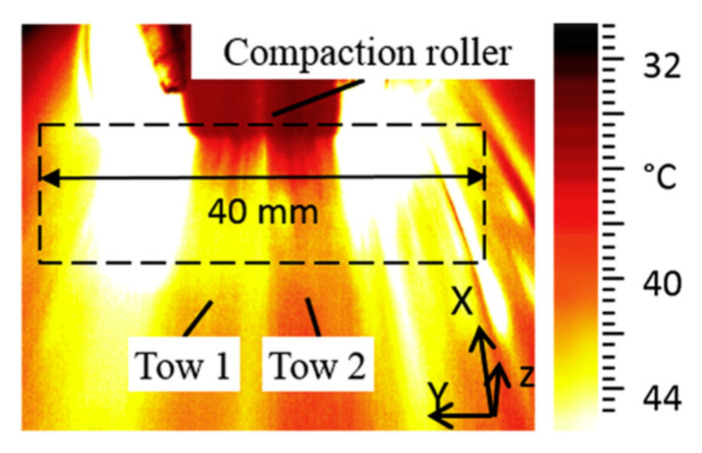
Thermal image of the tow and surrounding surface during the layup process [32].

Denkena et al. [32] presented an online AFP process monitoring method based on a thermal camera with image processing. The method can analyze the visible temperature difference between the laid-up tow and its surface underneath to identify the type of defects such as gaps, overlaps, twisted tows, and bridging derived, as shown in Figure 11. Although the monitoring system can reduce the efforts of quality inspections and contribute to improving process reliability significantly, the system can only detect surface defects. Internal defects and their evolution in the laminates are not recognized online.

Further work [123] by this research team has been executed recently. They used convolution neural networks (CNNs) to classify the thermal images of the (Carbon Fiber Reinforced Plastics) CFRP material, which can identify several prepreg materials and different material defects during the AFP process. Similar work has also been performed in [124]. Zambal et al. proposed formulating AFP defect detection as an image segmentation problem that can be solved in an end-to-end fashion using artificially generated training data. The results showed that the method can scale well with new defect types and measurement devices and requires little real-world data for training.

**Figure 12:**
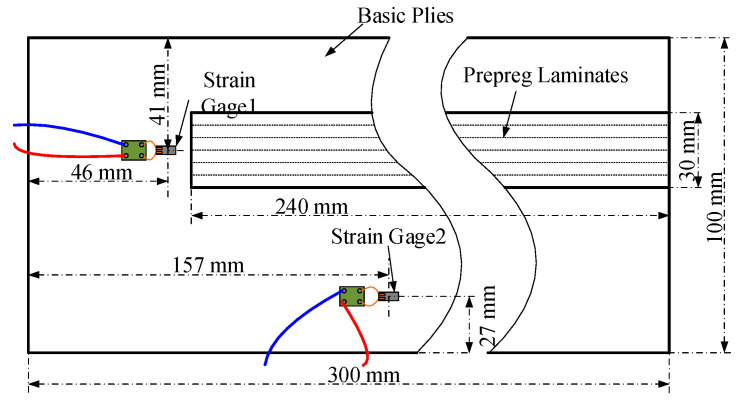
Test methods using the stress wave during the AFP process [111].

Stress waves can identify internal defects because the defects can change the characteristics of stress wave propagation. The stress wave has been widely used in non-destructive testing in the field of geology [125,126,127], tree damage diagnosis [128,129,130], and composites [131,132,133,134,135]. Han et al. [111] used the stress wave to detect online the internal defects during the AFP process, where continuous loading induced by the process itself was used as an excitation source without another external excitation (see Figure 12). The characteristics of stress waves, such as the amplitude, the Manhattan distance, and mean stress, were evaluated to obtain the relationship between the stress wave and the defects. But although this method can effectively identify the defect content, it cannot identify the type of defects.

Cemenska et al. [136] proposed an online detection method that integrates laser projectors, cameras, and laser profilometers in a comprehensive user interface, which can reduce the burden on inspectors and decrease run time.

Palardy-Sim et al. [137] described an innovative measurement solution based on optical coherence tomography (OCT), which can be easily integrated into the AFP head and allow measurements very close to the compaction roller nip point. The results showed that this inspection system can detect defects accurately.

**Figure 13:**
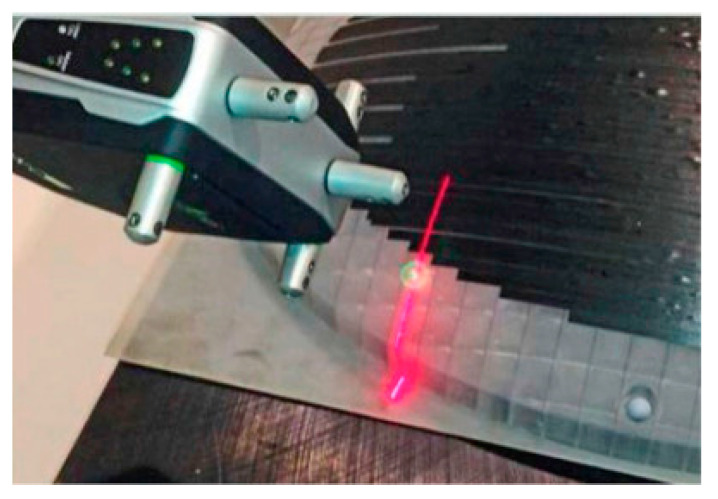
Laser line scanner detection process in the AFP process [139].

Krombholz et al. [138] presented a novel gap control method using a fiber edge detection sensor mounted on a CNC-controlled robot system, which can determine the relative positions of neighbored courses to allow a correction of the actual path, thereby controlling the gaps. This method has been used in the German Aerospace Center. Similarly, Maass et al. [139] described a commercial off-the-shelf (COTS) laser line scanner mounted on a robot or on an AFP head that can scan the projected laser line over the layup surface along a programmed path, which can be applied by NASA, as shown in Figure 13.

The literature on the online defect detection techniques in this review are summarized in Table 5.

## 5. Discussion and Conclusions

In summary, the types of position defects and their formation reasons during the AFP process have been thoroughly analyzed. The defects, such as gaps, overlaps, and twisted tows, can be induced by the combination of many factors, including machine tool errors, unreasonable process parameters, irrational angle planning, tow width limitation, and the in-plane bending deformation of a tow, etc. These defects may result in the formation of other defects. For instance, the gaps can lead to resin-rich areas or fiber waviness that affect the mechanical properties of composites. Moreover, the influence of defects on the mechanical properties of composites has been studied in depth, including the tensile, compression, shear, and even fatigue and vibration properties. However, not all defects hurt mechanical properties. For example, the overlaps can result in an absolute improvement in the compression strength. With this feature, we can avoid unfavorable defects as much as possible according to the different performance requirements of the parts or use favorable defects to reduce the impact of the unfavorable defects. The “overlap method” utilizes this principle, which can reduce the adverse effects of gap defects by controlling the translation distance to form overlaps between adjacent tows, but this method increases the thickness of the local regions. However, if the current review shows anything it is that the effects and interaction of gaps and overlaps in composite laminates is very complex, so we should be more cautious when this method is applied. This requires that, when a single defect is used to improve some performance, the complex formation mechanism of the defect and the interaction between the defects should be first understood thoroughly. To limit or control the effects of defects, researchers have proposed some measures to reduce defects, such as the tow-drop method, the staggering method, and CTS, but each has its scope and shortcomings. It seems that CTS is the most novel and promising control method, but the thickness of the parts at the current stage cannot be adjusted. Therefore, there have been few relevant studies on this topic in recent years. For engineering applications, the staggering method is still a relatively mature and effective means. From the research status of the type of prepregs, most of the studies focus on the laying defects of thermoset prepreg, but there are few studies on the defects of thermoplastic prepreg, in particular, thermoplastic materials with high-temperature resistance, such as PEEK and PPS matrix prepregs. It is well known that thermoplastic composites have the advantages of high toughness, high impact resistance and damage tolerance, unlimited storage, a short molding period, high production efficiency, easy repair, and recycling compared to traditional thermoset materials. Thus, thermoplastic materials are potential future aerospace materials. It can be seen that the study of the position defects of thermoplastic prepreg during the AFP process could become the focus of future research.

The formation mechanisms of void or bubble defects are entirely different compared to general defects such as gaps, overlaps, and wrinkles, etc. (Bubbles during the laying process are the potential causes of the formation of voids in composites.) Possible causes of induced bubbles include air inclusion during the laying process (this may be related to micro-wrinkles), gas volatilization during the laying or curing process, prepreg bubbles, and low manufacturing pressure, etc. Although it appears that the formation of voids is more closely related to the curing process, the effects of the laying process on the motion characteristics of bubble formation, splitting, and confluence, etc., cannot be neglected. Most current publications have focused on the optimization of the curing process or the effect of voids on the mechanical properties of cured composites. However, for thermoset materials, there is a correlation between bubbles before curing and voids after curing. For the in-situ curing of thermoplastic materials, the laying process directly determines the formation and distribution of voids. Therefore, studying the relationship between the behavioral characteristics of the bubbles and the process parameters, and the relationship between the bubbles during the laying process and the final voids after curing, is of great benefit to further improve the mechanical properties of the composites.

At this stage, online detection techniques for laying defects during the AFP process include machine vision, digital image processing, thermographic monitoring, and optical sensors, etc. However, machine vision, digital image processing, and thermographic monitoring can only detect surface defects. Although each ply can be detected during the AFP process, for large-scale workpieces this method needs to deal with a large amount of data. Additionally, the evolution of internal defects caused by multiple pressures during the AFP process cannot be detected. Therefore, it is also feasible to detect internal defects in each manufacturing stage using optical sensors and physical waves. The optical fiber Bragg grating sensor has been studied, but it needs to be embedded in the uncured laminate. This is a challenge for taking out the sensor and then curing. Stress waves and the online ultrasonic detection of defects are novel ways to detect the distribution of internal defects. However, the type of defects cannot be identified. Additionally, the relationship between process parameters and defect behavior has not been evaluated, so a reasonable process-based defect control strategy cannot be established. Therefore, combining different sensors with their various advantages to develop a multi-sensor online defect detection system and then embedding a reasonable process control strategy will become the focus of studying the online detection and controlling defects in the future.

## Figures and Tables

**Table 1:** Performance comparison of different defect configurations ^1^ [35].

		Gap	Overlap	Half Gap/Overlap	Twisted Tow
Tension		—	—	↓	↑
Compression		—	↑	—	—
In-plane shear	Length	—	—	↓	↓
	Width	↑	↓	↓	↓
OHT		—	—	—	o
OHC	Length	↑	↑	↑	o
	Width	↓	↓	↓	o

^1^ Note: ↑ refers to ≥3% increase, ↓ refers to ≤3% decrease, — refers to ±3% variation, o represents no test. OHT = open hole tension; OHC = open hole compression.

**Table 2:** The works on the relationships between position defects and mechanical properties in conventional laminates.

Contributors	Defects				Methods	Mechanical Properties
	Gaps	Overlaps	Fiber Waviness	Twisted Tow		
Sawicki, et al. [33,34]	+	+			Experiments	Compression strength
Croft, et al. [35]	+	+		+	Experiments	Tension, compression, in-plane shear, OHT and OHC
Marrouze, et al. [36]	+		+		Simulations experiments	Strength and stability
Lan, et al. [37]	+	+			Experiments (embedded defects)	Compression and in-plane shear
Guin, et al. [38]	+				Experiments	Tension, compression and OHC
Woigk, et al. [39]	+				Experiments	Tension and compression
Elsherbini, et al. [44]	+				Experiments	Fatigue

**Table 3:** Remote failure stress for un-notched tensile (UNT) and open-hole tensile (OHT) specimens [67].

Configurations	Mean Strength (MPa)	Standard Deviation (MPa)	Normalized Strength	Defect Area (%)
UNT baseline	389.2	0.6	1	-
UNT 100% coverage	347.3	12.3	0.89	2.38
UNT 0% coverage	303.1	21.7	0.78	2.38
UNT staggering (0% coverage)	355.8	9.1	0.91	2.38
OHT baseline	225.6	4.2	1	-
OHT 100% coverage	235.9	6.8	1.04	2.24
OHT 0% coverage	214.7	1.4	0.95	2.24
OHT staggering (0% coverage)	231.4	6.0	1.02	2.24

**Table 4:** The works on the defect control method and the relationships between position defects and mechanical properties in variable-stiffness laminates.

Contributors		Defects			Methods	Mechanical Properties
		Gaps	Overlaps	Others		
Control method	Blom et al. [52]	+	+		Tow-drop method	Strength and stiffness
	Blom et al. [59]	+	+		Staggering method	Structural performance
	Kim et al. [60,61,62,63]	+	+		CTS method	—
Mechanical properties	Fayazbakhsh et al. [64]	+	+		Finite Element Method (FEM)	In-plane stiffness and bulking load
	Wu et al. [65]	+	+		Experiments	Bulking stiffness
	Nik et al. [66]	+	+		Pareto solutions	In-plane stiffness and bulking load
	Falco et al. [67]	+			Experiments	UNT and OHT
	Falco et al. [68]	+			Meso testing, FEM	In-plane tensile
	Li et al. [69]	+	+		Modeling	Out-of-plane waviness
	Bakhshi et al. [83]			+	Modeling	—
	Akbarzadeh et al. [89]	+			Simulations, experiments	Vibration

**Table 5:** The literature on the online defect detection techniques.

Contributors	Detection Methods	Detectable Defects	Defect Type	Applications
Oromiehie et al. [122]	Optical fiber Bragg grating sensors	Gaps, overlaps	Internal defects	Laboratory
Denkena et al. [32]	Thermal camera with image processing	Gaps, overlaps	Surface	—
Schmidt et al. [123]	Convolution neural networks	Gaps, overlaps	Surface	—
Zambal et al. [124]	Artificially generated training data	Gaps, overlaps	Surface	—
Han et al. [111]	Stress wave	Voids	Internal defects	Laboratory
Cemenska et al. [136]	Laser projectors, cameras, and laser profilometers	Gaps, overlaps	Surface	—
Palardy-Sim et al. [137]	Optical coherence tomography	Gaps, overlaps, voids	Surface and internal defects	The National Research Council of Canada
Krombholz et al. [138]	Fiber edge detection sensor	Gaps, overlaps	Surface	The German Aerospace Center
Maass et al. [139]	Laser line scanner	Gaps, overlaps	Surface	NASA
